# Glycolysis at 75: is it time to tweak the first elucidated metabolic pathway in history?

**DOI:** 10.3389/fnins.2015.00170

**Published:** 2015-05-15

**Authors:** Avital Schurr, Evelyne Gozal

**Affiliations:** ^1^Department of Anesthesiology and Perioperative Medicine, School of Medicine, University of LouisvilleLouisville, KY, USA; ^2^Department of Pediatrics, School of Medicine, University of LouisvilleLouisville, KY, USA; ^3^Department of Pharmacology and Toxicology, School of Medicine, University of LouisvilleLouisville, KY, USA

**Keywords:** energy metabolism, glycolysis, lactate, lactate dehydrogenase, cancer, traumatic brain injury, pyruvate, monocarboxylate transporters

The sequence of the glycolytic enzymatic reactions responsible for the breakdown of glucose into two trioses and further into pyruvate or lactate was elucidated in 1940. For over seven decades it has been taught precisely the way that sequence was first described by Embden, Meyerhof and Parnas. Accordingly, two different glycolytic outcomes were proposed; the aerobic one with pyruvate as the final product, and the anaerobic one, identical to aerobic glycolysis except for one additional step, the reduction of pyruvate to lactate. While pyruvate has been presented as the mitochondrial substrate of the tricarboxylic acid cycle, lactate has regularly been vilified as a useless and frequently toxic end product of anaerobic glycolysis. Several studies in the 1980s have shown that both muscle and brain tissues are capable of oxidizing and utilizing lactate as an energy substrate, thus challenging the monocarboxylate's ill reputation. Expectedly, these findings were met with great skepticism and doubts that lactate could play any role in bioenergetics. Despite many studies from the 1920s and 1930s that have clearly showed the ability of brain tissue to oxidize lactate, even the investigators who published them assumed that lactate oxidation was simply a clearing mechanism of this end-product of anaerobic glycolysis (Schurr, 2006, 2014). Nevertheless, in the past two decades monocarboxylate transporters (MCTs) were identified and localized in both cellular and mitochondrial membranes and the existence of a lactate receptor in the brain has also been suggested (Bergersen and Gjedde, 2012). Both direct and indirect evidence now indicate that the enzyme lactate dehydrogenase (LDH) resides not only in the cytosol, as part of the glycolytic pathway machinery, but also in the mitochondrial outer membrane. The mitochondrial form of the enzyme oxidizes lactate to pyruvate and concomitantly produces the reducing agent NADH (Passarella et al., 2014; Rogatzki et al., 2015). These findings have shed light on a major drawback of the originally proposed aerobic version of the glycolytic pathway i.e., its inability to regenerate NAD^+^, in contrast to the anaerobic glycolysis that features a cyclical ability to regenerate NAD^+^upon pyruvate reduction to lactate by the cytosolic LDH. The malate-aspartate shuttle (MAS), a major redox shuttle in the brain was proposed as an alternative pathway for NAD^+^ generation for aerobic glycolysis. However, the necessity for MAS to fulfill such function could be questioned if the glycolytic pathway always proceeded to its end-products, lactate and NAD^+^, or when the suggested alternative, a lactate-malate-aspartate shuttle (Kane, 2014) is considered. An additional dilemma the originally proposed aerobic glycolysis presents, has to do with the glycolytic pathway of erythrocytes, which despite its highly aerobic environment always produces lactate as its end-product. Meanwhile, several other functions have been suggested for lactate (Chambers et al., 2014; Galow et al., 2014; Hertz et al., 2014; Brooks and Martin, 2015; Goodwin et al., 2015). For decades, pyruvate has always been the substrate of choice when measuring the rate of mitochondrial State III respiration employing an oxygen electrode. However, only recently it has been shown that lactate can be used as a substrate, too (de Bari et al., 2008; Passarella et al., 2008, 2014). Additional experiments could be designed to provide further insight into the role of lactate in energy metabolism. For example, would the addition of mitochondria to ruptured red blood cells switch their glycolytic pathway to produce pyruvate instead of lactate?

This Research Topic, now published as an E-book, includes 14 contributions from investigators conducting research in this field who review, opine, comment, or publish original data. In their perspective, Mariga et al. (2014) elaborate on the role of lactate in cerebral malaria (CM). Based on the data available, the authors suggest that monocarboxylate transporters (MCTs) and the lactate receptor GPR81 could become novel therapeutic targets in CM. Hertz et al. (2014), in their article, review the different roles lactate plays in brain tissue, including the modulation of glucose utilization rate, diagnosis of brain-injured patients and mediation of redox and receptor signaling, memory, and gene transcription. While these authors are listing multiple roles for lactate, mainly involving gap junction-coupled astrocytes, lactate's direct role as an oxidative energy substrate is not on the list. In his contribution, Schurr (2014) has reviewed a great number of papers on muscle and brain energy metabolism that werepublished in the waning years of the 19th century and the first four decades or so of the 20th century, providing a historical perspective to narrate the evolution of thinking and research in this field. These studies were crucial in the formation of the dogma according to which lactate, the end-product of anaerobic glycolysis, is useless and at times, poisonous. Using Margolis's (1993) concept that “habits of mind” govern scientific beliefs, Schurr argues that habits of mind still influence many scientists' beliefs today pertaining to lactate uselessness and the dissociation of glycolysis into aerobic and anaerobic pathways. These “habits of mind” persist, despite data already available in the 1920s and 1930s demonstrating that the brain oxidizes lactate very efficiently. The contribution of glycogen in supporting axon conduction both in peripheral and central nervous systems, and the role lactate plays in it, is reviewed by Chambers et al. (2014). The authors suggest that the presence of significant steady concentration of lactate in the periphery of both central white matter and peripheral nerve under unstimulated baseline conditions indicates a continuous efflux of lactate to the interstitium. They argue for the reexamination of the “on demand” shuttling of lactate between cellular elements based on the existence of those lactate pools, and thus, offer a continuous lactate efflux surplus available for immediate neural requirements. In a perspective on lactate oxidation at the mitochondria, Kane (2014) put forward the idea that shuttled lactate from the cytosol to the mitochondrion operates in a manner very similar to the malate-aspartate shuttle, the purpose of which has been proposed to be the oxidation of lactate and a mitochondrial electron shuttle. Moreover, he proposes that the two shuttles, the lactate one and the malate-aspartate one, are necessarily interconnected. Passarella et al. (2014) opine on the continuous debate among scientists as to whether or not L-lactatedehydrogenase (L-LDH) is localized in mitochondria. The debate goes on despite the overwhelming evidence supporting the localization of mitochondrial L-lactatedehydrogenase (m-L-LDH) inside these organelles. The authors argue that m-L-LDH can be detected in the mitochondrion when isolation and purification procedures are carried out carefully such that mitochondria stay coupled. Several measurements and assays can be easily and persuasively be performed to ascertain the metabolism of lactate in mitochondria. Goodwin et al. (2015), in an opinion article, present a concise overview of some recent developments in the field of lactate metabolism and cancer. Summarizing their overview, the authors conclude that lactate is both a potent fuel oxidatively and a signaling molecule involved in angiogenesis, and that it can be generated and exported or imported by tumors. They propose that lactate-protected hypoglycemia may be a viable strategy in tumors that exhibit high lactate production despite adequate tissue oxygen tension, while monocarboxylate transporter inhibitors could be useful against tumors whose angiogenesis is driven by lactate. In another opinion article, Deitmer et al. (2015) provide a perspective on the role of lactate as a signaling molecule and a modulator of metabolic processes due to its cotransport with H^+^ via monocarboxylate transporters (MCTs). Accordingly, lactate transporters form a “transport metabolon” with carbonic anhydrases. Allaman et al. (2015) review the existing knowledge on the cerebral glyoxalase system in both astrocytes and neurons. These two cerebral cell types with their high glycolytic activity presumably produce the highly reactive dicarbonyl compound, methylglyoxal, as a by-product of glycolysis. Methylglyoxal is associated with pathologies such as diabetes and aging, although its neurotoxicity in the brain is not well characterized. The authors advance a concept attributing astrocytes and neurons differential adaptive glyoxalase defense mechanisms against methylglyoxal-induced cellular damage. Brooks and Martin (2015) review theirs and others' studies on the post traumatic brain injury (TBI) treatment. In their studies, the authors compared dextrose + insulin treatment to exogenous lactate infusion in TBI patients with intact hepatic and renal functions, demonstrating that the latter results in normal glycemia. Carpenter et al. (2015) also review the role of lactate in TBI and the possible beneficial effect of intravenous exogenous lactate supplementation to TBI patients. Zilberter et al. (2015) opine in their paper on the potential of pyruvate, the other glycolytic monocarboxylate, as the treatment for signature characteristics of many neurological diseases i.e., hypometabolism, oxidative stress, and neuroinflamation. The authors believe that pyruvate is an ideal candidate for the treatment of these pathologies due to its unique combination of neuroprotective properties.

In light of the wealth of information already available, as can be gleaned from the present compilation of papers about lactate and its central role in energy metabolism in most, if not in all, tissues and specifically in the brain, it is clearly the time to reexamine theoriginal dogma of glycolysis. The separation of glycolysis into two distinct pathways, aerobic and anaerobic, is outdated and misleading. The first metabolic pathway to be elucidated 75 years ago should be redrawn as a singular pathway, its substrate being glucose and its end-product being lactate, the ultimate substrate of the mitochondrial TCA cycle.

## Conflict of interest statement

The authors declare that the research was conducted in the absence of any commercial or financial relationships that could be construed as a potential conflict of interest.
